# Characterization and phylogenetic analysis of the complete plastome of *Amaranthus retroflexus* L. (Amaranthaceae), an annual weeds

**DOI:** 10.1080/23802359.2021.1970640

**Published:** 2021-09-06

**Authors:** Ben-Xia Lou, Shou-Jin Fan

**Affiliations:** College of Life Sciences, Shandong Provincial Key Laboratory of Plant Stress Research, Shandong Normal University, Ji’nan, Shandong, China

**Keywords:** *Amaranthus retroflexus*, plastome, phylogeny

## Abstract

The complete plastome of *Amaranthus retroflexus* L., a field weed, was identified in this study. The genome size was 150,710 bp and consists of a large single-copy (LSC: 83,892 bp) region, a small single-copy (SSC: 18,100 bp) region, and two inverted repeats (IRs: 24,359 bp) regions. GC content was 36.6%. A total of 113 genes were identified, including 79 protein-coding genes, four rRNA genes, and 30 tRNA genes. Twenty chloroplast genomes from Amaranthaceae were selected to reconstruct phylogenetic tree and the result supported that *A. retroflexus* was sister to *A. hypochondriacus* and *A. caudatus*.

*Amaranthus retroflexus* L. is a monoecious annual herb within Amaranthaceae, widely distributed all over the world. *A. retroflexus* is native to North America, it is dramatically expanded its distribution throughout the China for the past decades (Weber et al. 2008). This weed is a prolific seed producer and seriously endangers the growth of crops in farmland (Francischini et al. 2014). The *A. retroflexus* increasingly develop resistance due to extensively use of herbicides (Robertson 1985; Li et al. 2004; Powles and Yu 2010). This study reported the *A. retroflexus* complete plastome, which would provide a fundamental genetic resources for weeds prevention and analyzing its phylogenetic position.

Fresh leaves of *A. retroflexus* were collected from Changdao District (Shandong, China; 37°91′ N, 120°73′ E). A specimen was deposited at Herbarium of College of Life Sciences, Shandong Normal University (Shou-Jin Fan, Email: fansj@sdnu.edu.cn) under the voucher number 20120. Total genomic DNA was extracted using a modified CTAB method (Zhang et al. 2019; Guo et al. 2020). The library preparation and paired-end (PE) sequencing of total genomic DNA were conducted by the Illumina Novaseq platform at Novogene (Beijing, China). Organelle Genome Assembler (OGA, https://github.com/quxiaojian/OGA) was used to do plastome assembling. Annotation was accomplished with Plastid Genome Annotator (PGA, https://github.com/quxiaojian/PGA) (Qu et al. 2019). Manual annotation correction was performed through Geneious v9.1.4 (Kearse et al. 2012). In order to determine the phylogenetic position of *A. retroflexus*, a maximum-likelihood (ML) tree was reconstructed by RAxML v8.2.10 (Stamatakis 2014) using 1000 bootstrap replicates with GTRCAT model based on 73 protein-coding genes after alignment using MAFFT v7.313 (Katoh and Standley 2013).

The complete plastome of *A. retroflexus* (GenBank accession number: MW646089) was 150,710 bp in length, and comprised a large single-copy (83,892 bp) region, a small single-copy (18,100 bp) region, and a pair of inverted repeats (IRs, 24,359 bp) regions. The GC content of this plastome was 36.6%. The GC content of IR regions is 42.6%, higher than LSC (34.5%) and SSC (30.2%) regions. A total of 113 unique genes were encoded, including 79 PCGs, 30 tRNAs, and four rRNAs. There are genes with two copies, including *ndh*B, *rpl*2, *rpl*23, *rps*12, *rps*7, *rrn*16, *rrn*23, *rrn*4.5, *rrn*5, *trn*A-UGC, *trn*I-CAU, *trn*L-CAA, *trn*N-GUU, *trn*R-ACG, *trn*V-GAC, and *ycf*2. The ML phylogenetic tree showed that *A. retroflexus* was sister to *A. hypochondriacus* and *A. caudatus* (Figure 1). In conclusion, the plastome of *A. retroflexus* provides significant DNA molecular data for further phylogenetic and evolutionary analysis for *Amaranthus*.

**Figure 1.**
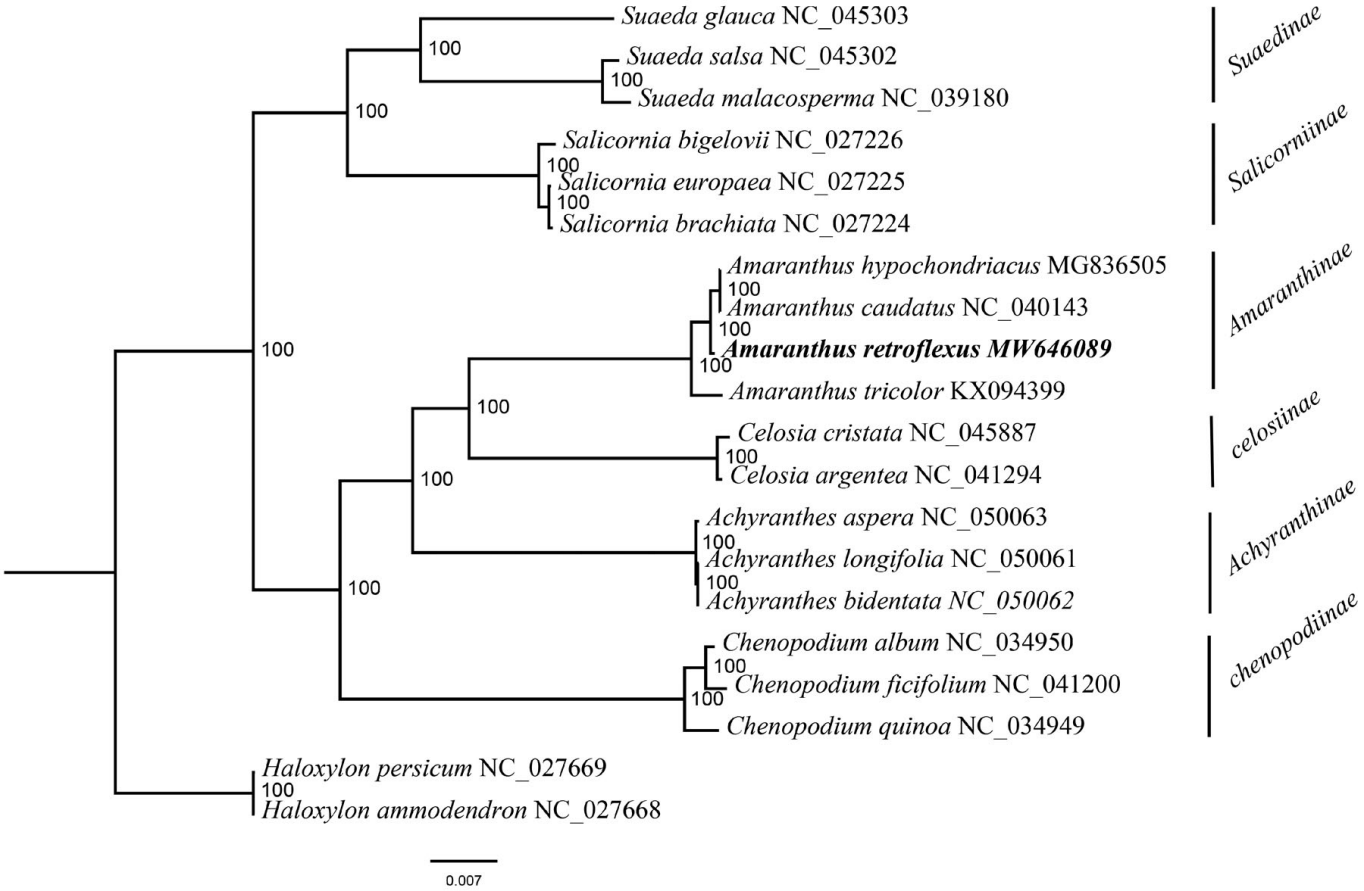
A maximum-likelihood (ML) phylogenetic tree based on 20 Amaranthaceae species is shown. Bootstrap support values are shown as numbers next to branches.

## Data Availability

The data that support the findings of this study are openly available in GenBank of NCBI, reference number MW646089. The associated BioProject, SRA, and Bio-Sample numbers are PRJNA718096 (http://www.ncbi.nlm.nih.gov/bioproject/718096), SRR14089437 (https://www.ncbi.nlm.nih.gov/sra/PRJNA718096), and SAMN18522283 (https://www.ncbi.nlm.nih.gov/sra/PRJNA718096), respectively.
